# Prevalence and factors associated with hepatitis B in a cohort of HIV-infected children in the Pediatric Department at Donka National Hospital, Guinea

**DOI:** 10.11604/pamj.2019.34.182.16275

**Published:** 2019-12-06

**Authors:** Djiba Kaba, Mmah Aminata Bangoura, Mariame Moustapha Sylla, Fodé Bangaly Sako, Mariama Sadjo Diallo, Issiaga Diallo, Ouo-Ouo Yaramon Kolié, Ahmed Sékou Keita, Boh Fanta Diané, Fatimata Keita, Mamady Diakité, Mamadou Diouldé Kanté, Moussa Savané, Djibril Sylla, Amadou Kaké, Kadiatou Diallo, Mafoudia Touré, Mohamed Cissé

**Affiliations:** 1 Université Gamal Abdel Nasser de Conakry, Conakry, Guinée; 2 Laboratoire de Biologie Moléculaire Nestor Bangoura/Hélène Labrousse, CTA Hôpital National Donka, Conakry, Guinée; 3 Service de Pédiatrie de l’Hôpital National Donka, Conakry, Guinée; 4 Service des Maladies Infectieuses et Tropicales de l’Hôpital National Donka, Conakry, Guinée; 5 Service de Dermatologie-Vénéréologie, Hôpital National Donka, Conakry, Guinée; 6 Service des Urgence Médico-Chirurgicales, Hôpital National Donka, Conakry, Guinée; 7 Service de Diabétologie et des Maladies Métaboliques, Hôpital National Donka, Conakry, Guinée; 8 Service de Médecine Interne, Hôpital National Donka, Conakry, Guinée

**Keywords:** Hepatitis B, HIV, prevalence, paediatrics, Donka National Hospital, Guinea

## Abstract

**Introduction:**

children pay a heavy price for infection with the hepatitis B virus (HBV). The objective of this work was to determine the prevalence of hepatitis B and describe the associated factors in children at the pediatric department of Donka Hospital.

**Methods:**

this was a cross-sectional study of a cohort of children in the pediatric department of Donka Hospital. HBsAg was performed by using an immunochromatographic method. The analysis of the data was done with software R. The proportions were compared using the Chi-square test or the Fisher test at the significance level of 5%. A logistic regression model was used to explain the prevalence of hepatitis B.

**Results:**

one hundred and forty-nine children were recruited between February and July 2017. HBsAg was present in 12 children, i.e. 8.16% (95% CI: 4.29-13.82). The average age was 93.32 months (IQR: 6-180). Male children were the most affected (n = 11, P <0.05), with a sex ratio of 1.01. The majority (51.35%) were on AZT + 3TC + NVP pediatric form and 25% were on AZT + 3TC + NVP adult form and 23.65% on TDF + FTC + EFV. In univariate analysis, ALT, HBsAg positivity, and maternal HBV vaccination status were associated with the prevalence of HBsAg (P <0.05).

**Conclusion:**

the prevalence of co-infection in children and adults is almost identical in our context. Hence the importance of strengthening preventive measures at all levels, especially the vaccination of children and mothers.

## Introduction

Hepatitis B virus (HBV) infection and human immunodeficiency virus (HIV) infection are considered by the World Health Organization (WHO) as major public health problems. HBV infection is the leading cause of acute and chronic liver disease in the world, such as cirrhosis or hepatocellular carcinoma, which is responsible for the majority of liver cancers worldwide [1]. The prevalence of HBV infection in children is 1.3 worldwide; 80 to 90% of infected infants in the first year of life will have a chronic infection and 30 to 50% of children infected before the age of 6 will be chronically infected [2]. HBV shares with HIV certain pathways of contamination, favoring the possibility of co-infection. For example, the prevalence of HIV/HBV coinfection is high in some “at risk” populations, such as intravenous drug users, polytransfused individuals, and so on. Vertical transmission is the major pathway of HBV and HIV infection in children [3] According to new data in 2017, WHO reports that 325 million people are living with chronic hepatitis B infection [4], of which 1.2 to 4.9% of children are carriers of HIV. HBsAg and infected with HIV [5, 6]. Sub-Saharan Africa is a region with a high prevalence of hepatitis B infection where the prevalence of HIV co-infection and hepatitis B varies between 10% and 20% in West and Central Africa [7, 8]. The prevalence of HIV/HBV in children in KwaZulu-Natal (South Africa) was 13.0% [9], in Nigeria, it was 8.3% [10]. In Guinea, prevalence studies conducted on HIV-HBV coinfection concern the adult population and are estimated at 8.49% [7]. The purpose of this study was to determine the prevalence of hepatitis B and describe the associated factors in HIV-infected children who were followed in the pediatric department at Donka National Hospital.

## Methods

This was a 6-month cross-sectional study from 1^st^ February to 31^st^ July 2017 in the pediatric department of Donka National Hospital. Laboratory analyzes were performed at the Nestor Bangoura/Hélène Labrousse Molecular Biology Laboratory of the Ambulatory Treatment Center (ATC) at Donka National Hospital.

**Study population:** we included all children aged 0 to 15 regardless of gender and followed for HIV infection, of which at least one of the parents or guardians gave informed consent and agreed to conduct the HBsAg research for the child. All mothers whose children participated in the study were included after obtaining their informed consent regardless of age or even HIV status.

**Data collection:** we recorded the medical records of HIV-infected children on ART followed in the Donka pediatric department during our study period. A questionnaire was used to collect sociodemographic data, family history of hepatitis, previous vaccination against hepatitis B, concept of blood exposure, clinical and paraclinical data. HBsAg was investigated in the serum of the participants using the AgHBs Kits^®^ rapid test from Gemc Technology Group Limited. All participants in the study were referred to the laboratory for venous sampling on dry tubes. All samples were centrifuged to obtain serum for analysis.

**Variables studied:** HIV + HBV coinfection were the dependent variables studied, either coded binary variable (yes or not). It was yes when the search for hepatitis B surface antigen (HBsAg) is positive in the HIV-infected child. Exposure factors were: age, sex, educational level, blood transfusion, maternal history of HBV vaccination, mode of delivery, WHO clinical stage, ALT, type of HIV, the most recent CD4 count, ART and the molecules used.

**Data processing and analysis:** regularly verified and corrected data were entered into Excel and analyzed using software R. Anonymity was respected by the use of the patient file number. The control of the database was done as the seizure progressed. The analysis first focused on the description of the socio-demographic profile of the subjects included. Frequencies were estimated with their confidence intervals and averages with their standard deviations. The proportions were compared using the Chi-square test or the Fisher test at the significance level of 5%. A logistic regression model was used to explain the prevalence of hepatitis B.

## Results

From February to July 2017, we recruited 149 HIV-infected children in the pediatric department of Donka National Hospital. The mean age of the children was 91.30 (months) ± 46.95 (p = 0.71) with a sex ratio of 1.01. Children in school (69.12%) versus 30.87% out of school with p = 0.44. The mean LTCD4 + level was 274.77 ± 232.92 with a higher mean in the male sex with no statistically significant difference. Table 1 summarizes the characteristics of the children who participated in the study. The most commonly used combination therapy was AZT + 3TC + NVP in adult form (53.7%) followed by pediatric form (24.8%). TDF + FTC + EFV accounted for only 21.5% of prescriptions. The most represented age group for mothers was 26-35 years old, 83.33%. Of the mothers, 66.67% were married, 41.67% were out of school, 62.4% were vaccinated. The majority of women (93.3%) had vaginal delivery and only 6.7% had delivered by caesarean section. Twelve out of the 149 children studied had HBsAg, which corresponds to a prevalence of 8.16% (95% CI: 4.29-13.82). Of the mothers, 8.05% were carriers of HBsAg. Of the 142 women who gave birth vaginally, 10 were carriers of HBsAg versus 2 of those delivered by caesarean section, and all 10 would have transmitted HBV to their newborn. Among the factors studied in univariate analysis, the sex of the child (p = 0.003), the maternal vaccination status (p = 0.000), the positivity of the HBsAg in the mother (p = 0.000), the rate of LTCD4 + (p = 0.03) and ALT (p = 0.000) were associated with carriage of HBsAg in children (Table 2). In multivariate analysis, only ALAT was associated with the carriage of HBsAg (Table 3).

**Table 1:** Characteristic of the study population

Variables/Categories	Frequency (%)/Mean with Standard Deviation (SD)		Total N = 149	P-value
Gender	Female n = 74	Male n = 75	-	
Sex-ratio	1,01			
Age	91,90 ± 46,95	94,72 ± 45,87	93,32 ± 46,27	0,71
Instruction level				
Schooled	50 (48, 53%)	53 (51, 47%)	103 (69, 12%)	
Unschooled	24 (52, 17%)	22 (47, 83%)	46 (30, 87%)	0,44
LT CD4^+^ (cells/mm^3^)	461,79 ± 374,11	517,01 ± 402,52	274,77 ± 232,92	0,40

**Table 2:** Factors associated with HIV-HBV coinfection

Variables/Categories	AgHBs+ N=12(%)	AgHBs- N=137(%)	p-value
**Age of the child (Month)**			
<36	2(1,34)	10(6,71)	0,220
36-71	4(2,68)	33(22,15)	
72-119	2(1,34)	41(27,52)	
120-180	4(2,68)	53(35,57)	
**Gender of the child**			
Male	11(7,38)	64(42,95)	0,003
Female	1(0,67)	73(48,99)	
**CD4 of the child**			
<500 mm^3^	10(6,71)	70(46,98)	0,037
≥500 mm^3^	2(1,34)	67(44,97)	
**ALAT of the child**			
≤40	1 (0,67)	135(90,60)	0,000
40	11 (7,38)	2(1,34)	
**HBs vaccination status of the mother**			0,000
Yes	12 (8,05)	81 (54,35)	
No	0	56 (37,59)	
**HBsAg of mother**			0,000
Positive	12(8,05)	0	
Negative	0	137(91,94)	
**Childbirth**			0,10
Caesarean	2(1,34)	5(3,56)	
Vaginal delivery	10(6,71)	132(88,59)	
**Therapeutic combination used by the child**			
AZT+3TC+NVP (Child)	9(6,04)	71(47,65)	0,086
AZT+3TC+NVP(Adult)	2(1,34)	35(23,49)	
TDF+FTC+EFV	1(0,67)	31(20,81)	

**Table 3:** Factors associated with HIV-HBV coinfection in multivariate analysis

Variables/Categories	OR (IC 95%)	p-value
**Vaccination against Hepatitis B**		
**Yes**	0,04(0,00-7,86)	0,232
**No**	Reference	-
**Transfusion**		
**Yes**	6,53(0,07-563,45)	0,409
**No**	Reference	-
**ALAT**	1,50(1,10-2,06)	0,010

## Discussion

We performed a cross-sectional study in a cohort of children followed at the pediatric department of Donka National Hospital. This study estimated the prevalence of HIV-HBV coinfection in this pediatric population at 8.16% (95% CI: 4.29-13.82) and identified some associated factors. The cross-sectional nature of the study does not make it possible to establish the causal links between the identifying factors and the carriage of HBsAg. This prevalence is based on the search for HBsAg performed on an immunochromatographic test which has the limit not to detect cases of occult hepatitis, the prevalence of which is estimated to be 5% [11]. Another limitation would be the lack of antibody assay and failure to achieve the HBV viral load that could reduce the minimization of cases of occult hepatitis. Despite this, the figures obtained make it possible to take stock of the situation on co-infection in the pediatric population. This prevalence is not different from that found in other studies, such as in South Africa [9], Côte d'Ivoire [12] and Nigeria [10] with respectively 13.0%, 12.1% and 8.3%. On the other hand, the prevalence in Tanzania [6] is 1.2% and 3.18% in Senegal [13]. It denotes the extent of HIV-HBV coinfection in this pediatric population with an immature immune defense and also weakened by HIV infection as indicated by the mean of TCD4+ lymphocytes which were 274.77 ± 232, 92. The most represented age group for mothers was 26-35 years old, 83.33%. Most of the infected mothers were married at 66.67%, 41.67% of whom were out of school. In Burkina [14], Sangaré L *et al.* reported that 59.28% were married and 42.35% had no level of education. The maternal vaccination rate was 62.4%, of which 12 had HBsAg. HBsAg carriers were associated with maternal vaccination status (p = 0.000). Ideally, all women of reproductive age should be vaccinated to protect future births. Of the 142 women who gave birth vaginally, 10 had HBsAg versus 2 among cesarean births. The 10 women reportedly transmitted HBV to their fetuses either during intrauterine life or during delivery even if, in multivariate analysis, the mode of delivery was not associated with carriage of HBsAg in children. Among the factors studied, the transaminase level (ALT) was the only factor associated after multivariate analysis (p = 0.000). Other factors studied such as age, LTCD4+ level, educational level, blood transfusion were not associated with carriage of HBsAg. Our results show the protective effect of vaccination, OR = 0.04 (0.00-7.86) although this does not appear statistically significant (p = 0.2). With respect to the therapeutic combinations used, the combination AZT + 3TC + NVP adult form (53.7%) was the most prescribed followed by its pediatric form (24.8%). The combination of TDF + FTC + EFV accounted for 21.5% of the prescriptions and only one of the children carrying HBsAg was in this combination. This combination used in older adolescents should be included in the treatment regimen for children with HIV-HBV coinfection [3]. TDF and FTC have been shown to be active in HBV and therefore HBV is the first-line combination in people coinfected with HIV and HBV [3].

## Conclusion

The prevalence of HIV-HBV co-infection in children followed in the pediatric department was 8.16%, which is not very different from the numbers in adults. It was higher in boys than in girls, in subjects with LTCD4+ <500 cells/mm^3^ and in those with abnormal ALAT levels.

### What is known about this topic

- HBV infection is the leading cause of acute and chronic liver disease in the world;
- Eighty to 90% of infected infants in the first year of life will be chronically infected and 30% to 50% of infants infected before the age of 6 will be chronically infected;
- Vertical transmission is the major route of infection of HBV and HIV in children.

### What this study adds

- A prevalence of HBV-HIV coinfection in children estimated at 8.16% (95% CI: 4.29-13.82);
- A vaccination rate for mothers at 62.4% and therefore there is effort to provide;
- A focus on vertical transmission. All infected children are born to HIV-positive mothers for HBsAg.

## Competing interests

The authors declare no competing interests.
